# WISP-3 promotes angiogenesis in non-small cell lung cancer through p38/JNK-c-Jun-mediated PDGF-A upregulation

**DOI:** 10.7150/jca.127681

**Published:** 2026-03-25

**Authors:** En-Ming Chang, Syuan-Ling Lin, Ching-Yuan Cheng, Chih-Hsin Tang, Yu-Chen Chen, Chiang-Wen Lee, Chih-Yang Lin

**Affiliations:** 1 Department of Respiratory Care, Shin Kong Wu Ho-Su Memorial Hospital, Taipei City, Taiwan.; 2 Translational Medicine Research Center, China Medical University Hospital, Taichung, Taiwan.; 3 Graduate Institute of Biomedical Sciences, China Medical University, Taichung, Taiwan.; 4 Division of Chest Medicine, Shin Kong Wu Ho-Su Memorial Hospital, Taipei, Taiwan.; 5 Department of Pharmacology, School of Medicine, China Medical University, Taichung, Taiwan.; 6 Department of Medical Laboratory Science and Biotechnology, Asia University, Taichung, Taiwan.; 7 Chinese Medicine Research Center, China Medical University, Taichung, Taiwan.; 8 Translational Medicine Center, Shin Kong Wu Ho-Su Memorial Hospital, Taipei, Taiwan.; 9 Department of Orthopaedic Surgery, Chang Gung Memorial Hospital, Chiayi, Taiwan.; 10 Department of Respiratory Care, Chang Gung University of Science and Technology, Chiayi, Taiwan.; 11 Chronic Diseases and Health Promotion Research Center and Center for smart healthcare education and Drug Research and Development, Chang Gung University of Science and Technology, Chiayi, Taiwan.; 12 Department of Safety Health and Environmental Engineering, Ming Chi University of Technology, New Taipei City, Taiwan.; 13 Department of Medical Research, China Medical University Hospital, China Medical University, Taichung 404327, Taiwan.

**Keywords:** WISP-3, PDGF-A, lung cancer, angiogenesis

## Abstract

Angiogenesis is a pivotal process for tumor progression and metastasis in non-small cell lung cancer (NSCLC). However, the molecular mechanisms by which WNT1-inducible signaling pathway protein 3 (WISP-3) contributes to NSCLC angiogenesis remain poorly defined. This study investigated the role of WISP-3 in regulating pro-angiogenic signaling in lung adenocarcinoma (LUAD) cells. Conditioned medium from H1299 and A549 cells treated with recombinant WISP-3 (0-100 ng/mL) significantly and dose-dependently enhanced the tube formation of human umbilical vein endothelial cells (HUVECs). WISP-3 selectively upregulated platelet-derived growth factor A (PDGF-A) expression at both mRNA and protein levels in NSCLC cell lines, while other angiogenic factors remained unaffected. Notably, knockdown of PDGF-A using siRNA markedly abolished WISP-3-induced HUVEC tube formation, confirming PDGF-A as a critical mediator in this process. Mechanistically, WISP-3 rapidly triggered the phosphorylation of p38 and JNK signaling pathways. These activations led to the phosphorylation of the transcription factor c-Jun, which in turn promoted PDGF-A gene expression. Pharmacological inhibition of p38 (Adezmapimod), JNK (SP600125), or c-Jun (T-5224) effectively suppressed WISP-3-induced c-Jun activation, PDGF-A expression, and subsequent angiogenesis. Collectively, our findings identify a novel WISP-3/p38-JNK/c-Jun/PDGF-A signaling axis that drives vascular remodeling in NSCLC. Targeting WISP-3 or its downstream effectors may represent a promising therapeutic strategy for anti-angiogenic treatment in lung cancer.

## 1. Introduction

Lung cancer remains the leading cause of cancer-related mortality worldwide, with lung adenocarcinoma (LUAD) being the most common histological subtype, accounting for approximately 40-45% of all lung cancer cases 1. LUAD primarily arises from bronchial epithelial cells or mucous gland cells and exhibits highly invasive characteristics 2. While surgical resection remains the standard treatment for early-stage disease, advanced-stage LUAD requires multimodal therapies, including chemotherapy, radiotherapy, targeted therapy, and immunotherapy. Despite these clinical advances, the overall five-year survival rate remains below 20%, and recurrence after radical surgery is common 1. Growing evidence suggests that tumor angiogenesis within the tumor microenvironment (TME) is integral to the progression, invasion, and metastasis of LUAD 3. Anti-angiogenic therapies have demonstrated clinical benefits in advanced cases, underscoring the importance of vascular regulation in tumor biology 4. Therefore, elucidating the molecular mechanisms underlying angiogenesis and identifying key regulatory genes involved in LUAD vascular remodeling are essential for developing novel and more effective therapeutic strategies.

Among the key regulators of tumor angiogenesis, the platelet-derived growth factor (PDGF) family plays a pivotal role in vascular development and remodeling 5. The PDGF family consists of four ligands (PDGF-A, -B, -C, and -D) that function as homo- or heterodimers and signal through two tyrosine kinase receptors, PDGFRα and PDGFRβ 6. PDGF signaling promotes endothelial cell proliferation, pericyte recruitment, and stabilization of the microvasculature, processes essential for maintaining vessel integrity within tumors 5, 7. In the tumor microenvironment, aberrant activation of PDGF pathways enhances angiogenic signaling by inducing vascular endothelial growth factor (VEGF) expression and supporting stromal cell interaction 8. Specifically, tumor-derived PDGF-A and PDGFRα have been implicated in recruiting angiogenic stroma and stimulating the secretion of VEGF-A and other pro-angiogenic mediators 5. Dysregulation of this pathway contributes to tumor progression, metastasis, and resistance to anti-VEGF therapy 1, 6. Therefore, understanding how PDGF-driven angiogenic signaling operates in lung adenocarcinoma may reveal new molecular targets for anti-angiogenic intervention.

WNT1-inducible signaling pathway protein 3 (WISP-3), also known as CCN6, is a secreted matricellular protein belonging to the CCN family, which comprises six cysteine-rich growth regulators (CCN1-6) involved in extracellular matrix remodeling, cell adhesion, and angiogenesis 9. Structurally, WISP-3 contains four conserved domains that facilitate interactions with integrins, proteoglycans, and growth factors, thereby mediating critical crosstalk between tumor and stromal cells 10. Dysregulation of WISP-3 has been documented in various malignancies, including breast 11, chondrosarcoma 12, and gastrointestinal tumors 13, where it modulates signaling pathways such as PI3K/AKT, MAPK, and TGF-β to influence cell proliferation, migration, and invasion 10. Recent transcriptomic and proteogenomic analyses have identified CCN6 as an upregulated biomarker in LUAD and a key mediator of fibroblast activation and angiogenic remodeling within the tumor microenvironment 13. Specifically, elevated WISP-3 expression in the tumor stroma promotes fibroblast proliferation and enhances angiogenic potential 14. Emerging evidence further suggests that WISP-3 regulates the tumor microenvironment through paracrine modulation of pro-angiogenic factors, including PDGF and VEGF 15, thereby contributing to vascular remodeling and neovascularization in LUAD.

Despite increasing recognition of the CCN family in tumor biology, the precise role of WISP-3 in regulating angiogenesis in LUAD remains poorly defined. Considering the established functions of PDGF signaling in pericyte recruitment and vascular maturation, as well as preliminary evidence linking WISP-3 to fibroblast activation and angiogenic remodeling, we hypothesized that WISP-3 promotes LUAD angiogenesis through the transcriptional upregulation of PDGF-A via specific downstream signaling pathways. To test this hypothesis, we investigated the effects of recombinant WISP-3 on endothelial tube formation and examined its regulatory impact on PDGF-A expression in LUAD cells. We further explored the underlying molecular mechanisms, focusing on the p38/JNK-c-Jun signaling axis, and evaluated whether pharmacological inhibition or genetic knockdown of these pathways attenuates WISP-3-induced angiogenesis. This study aims to elucidate the mechanistic link between WISP-3 and PDGF-A-mediated vascular remodeling, thereby identifying WISP-3 as a potential pro-angiogenic driver and therapeutic target in lung adenocarcinoma.

## Materials and Methods

### Materials

The following antibodies and reagents were used: rabbit polyclonal anti-SAPK/JNK (Cat. No. 9295) from Cell Signaling Technology (Danvers, MA, USA); anti-p-p38 MAPK (E-1) (Cat. No. SC-166182), anti-p38α MAPK14 (F-9) (Cat. No. SC-271120), anti-p-JNK (G-7) (Cat. No. SC-6254), anti-p-c-Jun (KM-1) (Cat. No. SC-822), and anti-c-Jun (G-4) (Cat. No. SC-74543) from Santa Cruz Biotechnology (Santa Cruz, CA, USA); anti-PDGF-A (Cat. No. AF5179) from Affinity Biosciences (Jiangsu, China); and anti-β-actin (Cat. No. A5441) from Sigma-Aldrich (St. Louis, MO, USA). Cell culture reagents, including Dulbecco's Modified Eagle Medium (DMEM), Roswell Park Memorial Institute (RPMI)-1640, and fetal bovine serum (FBS), were purchased from Gibco-BRL Life Technologies (Grand Island, NY, USA). All other chemicals and reagents were purchased from Sigma-Aldrich unless otherwise specified.

### Cell lines and culture

The human NSCLC cell lines A549 and NCI-H1299 were procured from the American Type Culture Collection (ATCC, Manassas, VA, USA). These cells were maintained in RPMI-1640 medium supplemented with 10% FBS, 100 U/mL penicillin, and 100 μg/mL streptomycin, and cultured in a humidified incubator at 37°C with 5% CO_2_.

Human umbilical vein endothelial cells (HUVECs) (BCRC No. H-UV001) were obtained from the Bioresource Collection and Research Center (BCRC, Hsinchu, Taiwan) and maintained in DMEM supplemented with 10% FBS and antibiotics (100 U/mL penicillin and 100 μg/mL streptomycin) at 37 °C in a humidified atmosphere containing 5% CO₂ 16.

### Western blot analysis

Cell lysates were prepared as previously described 17, 18. Equal amounts of protein were resolved by sodium dodecyl sulfate-polyacrylamide gel electrophoresis (SDS-PAGE) and transferred onto polyvinylidene fluoride (PVDF) membranes. The membranes were blocked with Tris-buffered saline containing 0.1% Tween-20 (TBST) and 4% non-fat milk at room temperature for 1 h, then incubated with the indicated primary antibodies (anti-p-p38, p38, p-JNK, JNK, p-c-Jun, c-Jun, PDGF-A, and β-actin) for 1 h at room temperature or overnight at 4 °C. After three 5-min washes with TBST, the membranes were incubated with HRP-conjugated anti-rabbit or anti-mouse secondary antibodies for 1 h at room temperature and washed again three times with TBST. Protein bands were detected using an enhanced chemiluminescence (ECL) substrate (prepared according to the manufacturer's instructions) and visualized with a Fujifilm LAS-3000 imaging system (Fujifilm, Tokyo, Japan). Band intensity was quantified using UN-SCAN-IT gel 6.1 software, and normalized data were analyzed and plotted using GraphPad Prism 8.0.

### Quantitative real-time polymerase chain reaction (qRT-PCR)

Total RNA was extracted from A549 and H1299 lung cancer cells using the TRIzol reagent (MDBio Inc., Taipei, Taiwan). RNA purity was assessed using a NanoDrop ND-1000 spectrophotometer (Thermo Fisher Scientific, USA), with 260/280 nm ratios between 1.8 and 2.0 deemed acceptable. Complementary DNA (cDNA) was synthesized using the Invitrogen Reverse Transcription Kit (Carlsbad, CA, USA). qRT-PCR was performed in a 20 µL reaction containing 2 µL of cDNA, the respective primers, and SYBR Green PCR Master Mix (Thermo Scientific, Waltham, MA, USA). The primer sequences used for the targeted genes (PDGF-A, PDGF-B, PDGF-C, PDGF-D, VEGF-A, and GAPDH) are summarized in Table 1. Relative mRNA expression levels were calculated using the 2⁻ΔΔCt method and normalized to glyceraldehyde-3-phosphate dehydrogenase (GAPDH) as an internal control 17.

### Cell transfection

Small interfering RNAs (siRNAs) targeting PDGF-A, as well as a non-targeting control siRNA, were obtained from Santa Cruz Biotechnology (Santa Cruz, CA, USA). Transient siRNA transfection was performed using Lipofectamine 2000 (Invitrogen, Carlsbad, CA, USA) according to the manufacturer's instructions. LUAD cells were seeded in 6-well plates at a density of 5 × 10⁵ cells per well and cultured until approximately 80% confluence. Prior to transfection, the culture medium was replaced with serum- and antibiotic-free medium. Cells were then transfected with 100 nM target-specific siRNA or control siRNA and incubated for 24 h. Following transfection, cells and CM were harvested and subjected to quantitative PCR, Western blot analyses, and tube formation assays to evaluate gene silencing efficiency and downstream signaling effects, as previously described.

### Collection of lung cancer conditioned medium (CM)

To prepare CM, H1299 lung cancer cells were pretreated with pharmacological inhibitors as indicated, followed by WISP-3 treatment. The culture supernatants were collected, centrifuged to remove cellular debris, aliquoted, and stored at -80 °C.

### HUVEC tube formation assay and quantification

Matrigel (120 µL per well) was plated into a 48-well plate and polymerized at 37 °C for 30 min. HUVECs were seeded at a density of 3 × 10⁵ cells/well in a 1:1 mixture of EGM™-2MV medium and CM from lung cancer cells. After 6 h of incubation, tube formation was visualized and photographed using a light microscope. The total branch points were quantified from five random fields per well using ImageJ software.

### Statistical analysis

All experiments were performed in triplicate and data are presented as the mean ± standard deviation (SD). Statistical significance was assessed using a two-tailed Student's t-test for comparisons between two groups, with a threshold of p < 0.05 considered significant. All analyses and graph preparation were performed using GraphPad Prism 8.0 software.

## Results

### WISP-3 promotes angiogenic activity and induces PDGF-A expression in LUAD cells

**Figure 1:**
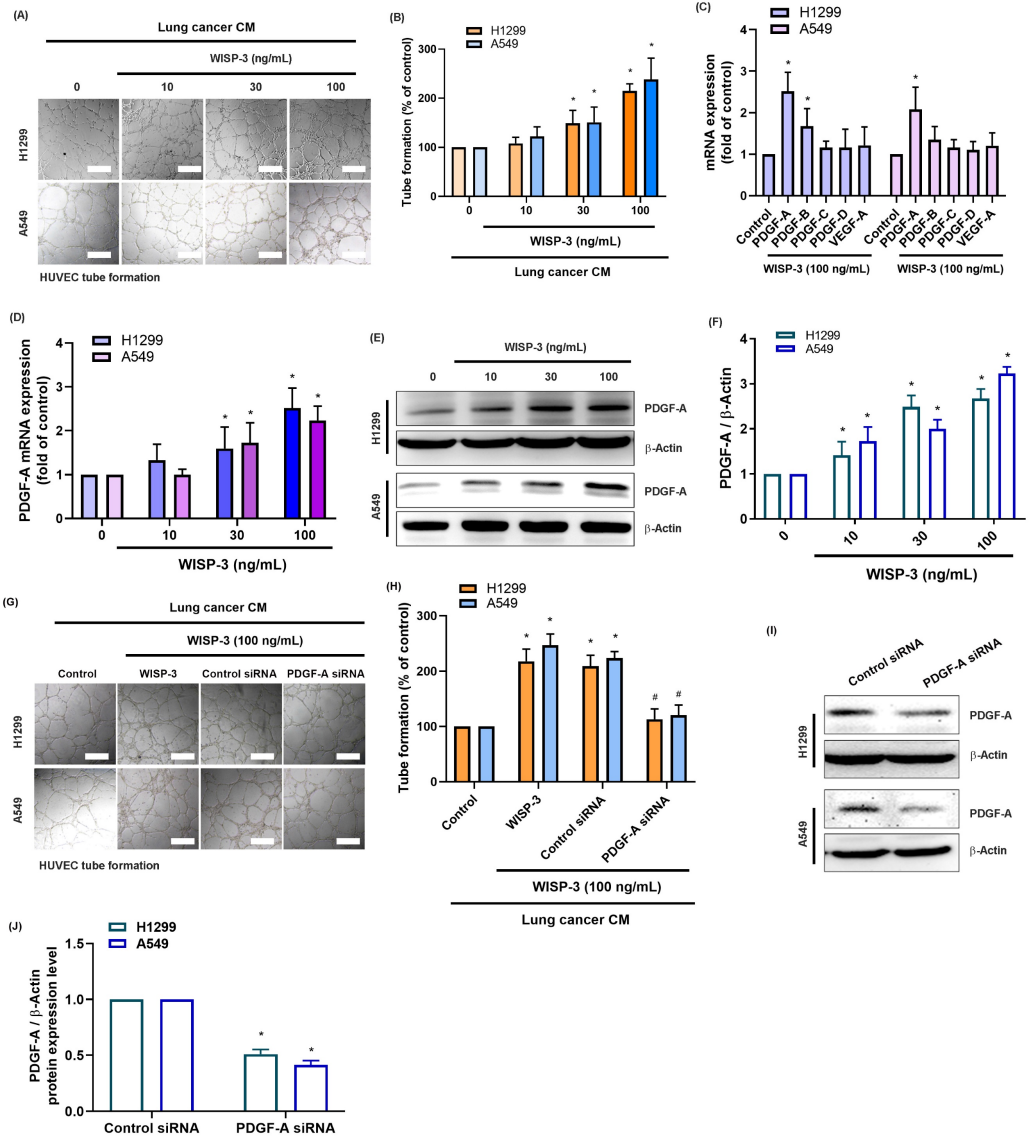
** WISP-3 promotes PDGF-A expression and enhances HUVEC tube formation in LUAD cells.** (A) Representative micrographs illustrating the tube formation of HUVECs cultured with CM from H1299 and A549 cells treated with increasing concentrations of recombinant WISP-3 (0-100 ng/mL). (B) Quantitative analysis of total tube branches relative to the control group. (C) qRT-PCR analysis showing the mRNA expression of PDGFA, PDGFB, PDGFC, PDGFD, and VEGFA in LUAD cells following WISP-3 treatment (100 ng/mL). (D) Relative PDGFA mRNA levels in H1299 and A549 cells stimulated with indicated concentrations of WISP-3. (E) Western blot analysis displaying PDGF-A protein expression in both LUAD cell lines. (F) Densitometric quantification of PDGF-A protein levels normalized to β-actin. (G) Representative images of HUVEC tube formation showing that the pro-angiogenic effect of WISP-3 (100 ng/mL) is abolished by PDGF-A siRNA transfection in H1299 and A549 cells. (H) Quantitative assessment of tube branch numbers following PDGF-A knockdown. (I) Western blot analysis confirming the knockdown efficiency of PDGF-A siRNA in both cell lines. (J) Densitometric quantification of PDGF-A protein levels following siRNA transfection. Data are presented as mean ± SD (n ≥ 3). *p < 0.05 vs. control; #p < 0.05 vs. WISP-3. Scale bar = 200 μm (for all micrographs).

Based on previous findings indicating that WISP-3 modulates cell proliferation, migration, and extracellular matrix remodeling in various cancers 19, 20, we first investigated its role in LUAD angiogenesis. Treatment of HUVECs with CM from H1299 and A549 cells stimulated with recombinant WISP-3 (0-100 ng/mL) significantly and dose-dependently enhanced tube formation (Fig. 1A&B). Subsequently, qRT-PCR analysis revealed that WISP-3 selectively upregulated PDGF-A mRNA, while the levels of other angiogenic factors (PDGF-B, PDGF-C, PDGF-D, and VEGF-A) did not change significantly (Fig. 1C). Consistently, WISP-3 increased PDGF-A expression in both H1299 and A549 cells in a concentration-dependent fashion at both the mRNA (Fig. 1D) and protein levels, as confirmed by Western blot analysis (Fig. 1E&F). To further verify the causal relationship between WISP-3-induced PDGF-A expression and angiogenesis, we utilized PDGF-A-specific siRNA to knockdown its expression. Our results showed that the genetic silencing of PDGF-A markedly abolished the WISP-3-induced increase in HUVEC tube formation (Fig. 1G&H). The knockdown efficiency of the siRNA was confirmed by Western blot analysis in both cell lines (Fig. 1I&J). Together, these findings demonstrate that WISP-3 enhances the angiogenic potential of LUAD cells specifically by inducing PDGF-A expression, identifying PDGF-A as a critical downstream effector of WISP-3 in tumor angiogenesis.

### WISP-3 activates the p38 and JNK signaling pathways to induce PDGF-A expression and angiogenesis

**Figure 2:**
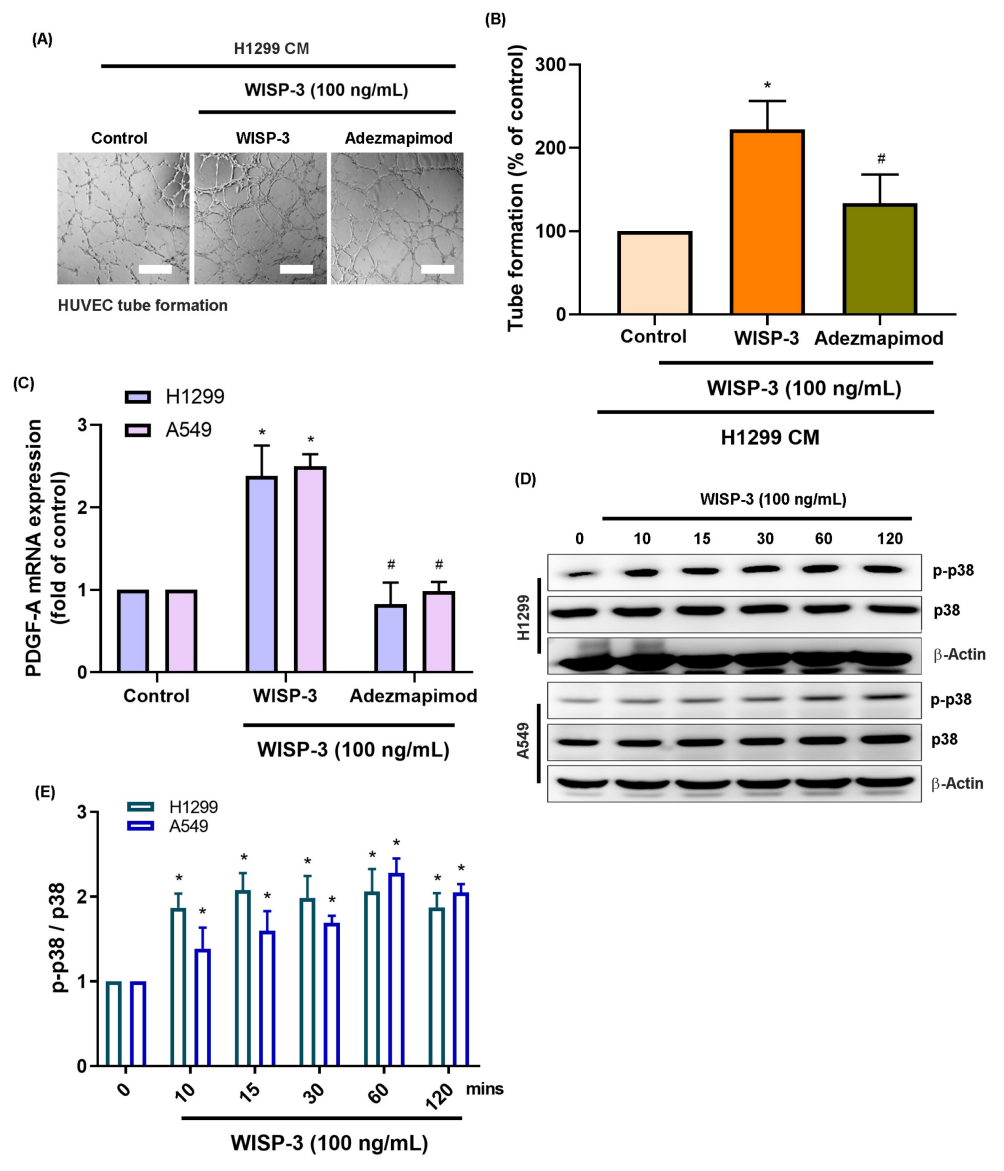
** Activation of the p38 signaling pathways is required for WISP-3-induced PDGF-A upregulation and angiogenic activity.** (A) Representative micrographs of HUVEC tube formation following treatment with CM from LUAD cells stimulated with WISP-3 (100 ng/mL) alone or in combination with the p38 inhibitor Adezmapimod. (B) Quantitative analysis of total tube branch numbers relative to the control group. (C) qRT-PCR results demonstrating that WISP-3-induced PDGF-A mRNA upregulation in H1299 and A549 cells is significantly attenuated by p38 inhibition. (D) Western blot analysis of time-dependent phosphorylation of p38 in both H1299 and A549 cells after WISP-3 stimulation (0-120 min). (E) Densitometric quantification of p-p38/p38 ratios, with data normalized to the control group. Data are shown as mean ± SD (n ≥ 3). *p < 0.05 vs. control; #p < 0.05 vs. WISP-3. Scale bar = 200 μm (for all micrographs).

**Figure 3:**
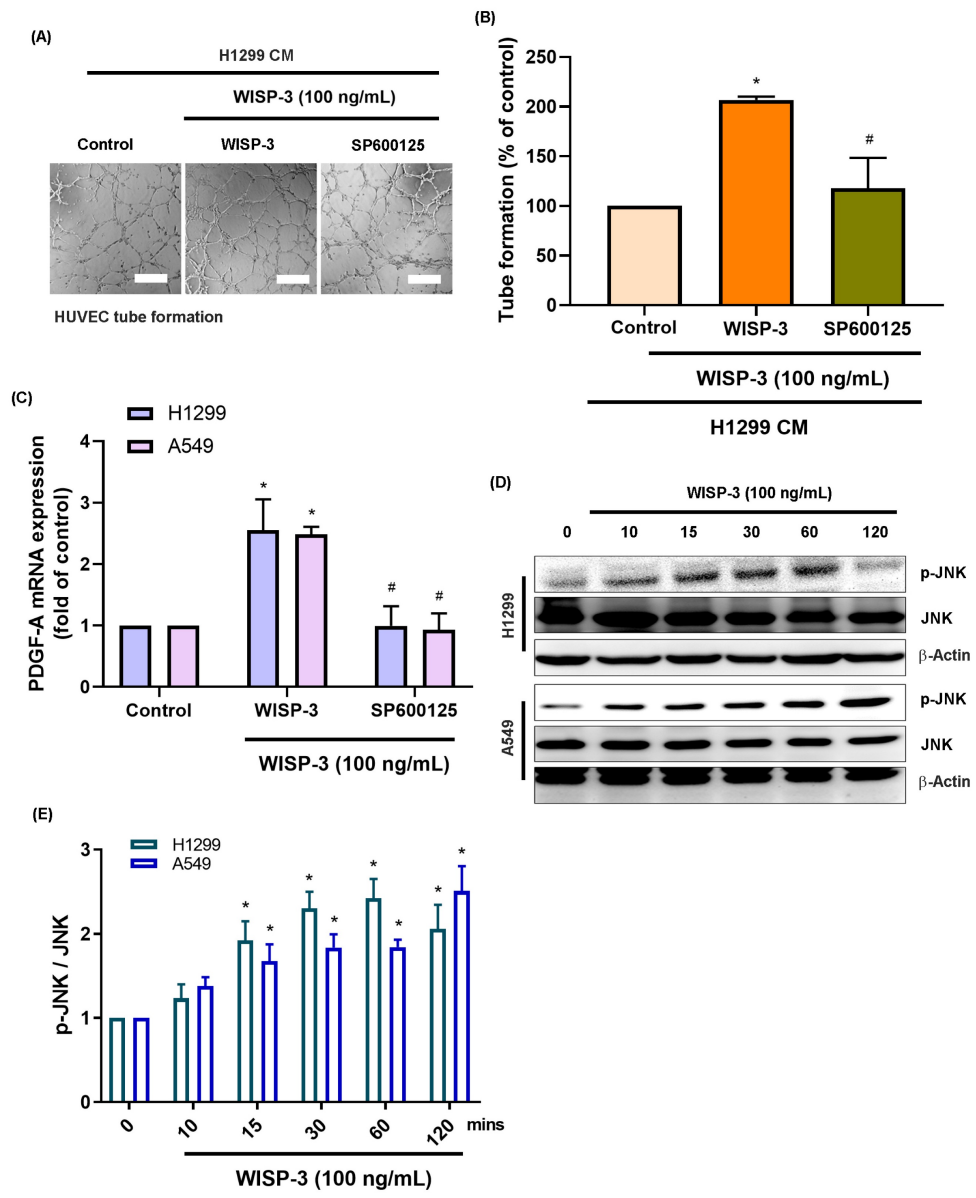
** Inhibition of JNK suppresses WISP-3-mediated PDGF-A expression and endothelial tube formation.** (A) Representative micrographs of HUVEC tube formation showing the reduced angiogenic ability of HUVECs exposed to WISP-3-treated CM in the presence of the JNK inhibitor SP600125. (B) Quantitative analysis of total tube branch numbers relative to the WISP-3 treatment alone. (C) qRT-PCR analysis of PDGF-A mRNA levels in LUAD cells under the indicated treatments. (D) Western blot analysis demonstrating decreased PDGF-A protein levels following pharmacological inhibition of JNK. (E) Densitometric analysis of p-JNK/JNK ratios, with data normalized to the control group. Data represent mean ± SD (n ≥ 3). *p < 0.05 vs. control; #p < 0.05 vs. WISP-3. Scale bar = 200 μm (for all micrographs).

Mitogen-activated protein kinases (MAPKs), specifically p38 and JNK, are recognized as key regulators of tumor angiogenesis by modulating endothelial migration and growth-factor signaling across various malignancies 21. Given that PDGF-A transcription can be regulated by AP-1 components downstream of these kinases, we investigated whether the p38 and JNK cascades mediate WISP-3-induced PDGF-A expression in LUAD cells. Consistent with our previous findings, treatment with recombinant WISP-3 (100 ng/mL) significantly enhanced HUVEC tube formation. However, pharmacological inhibition of p38 (Adezmapimod) or JNK (SP600125) markedly attenuated this pro-angiogenic effect (Fig. 2A&B and 3A&B). In both H1299 and A549 cells, WISP-3 stimulation upregulated PDGF-A mRNA was also suppressed by either inhibitor (Fig. 2C&3C). Western blot analysis further revealed that WISP-3 rapidly triggered the phosphorylation of both p38 and JNK, with activation peaking at 30-60 min and remaining elevated for up to 120 min (Fig. 2D&E and 3D&E). These results demonstrate that WISP-3 activates the p38 and JNK pathways, both of which are indispensable for the transcriptional upregulation of PDGF-A and the subsequent enhancement of angiogenic activity in LUAD. Collectively, these findings identify the p38/JNK axis as a critical signaling intermediary linking WISP-3 to PDGF-A-driven vascular remodeling.

### WISP-3 activates c-Jun via p38 and JNK signaling to regulate PDGF-A expression and angiogenesis

**Figure 4:**
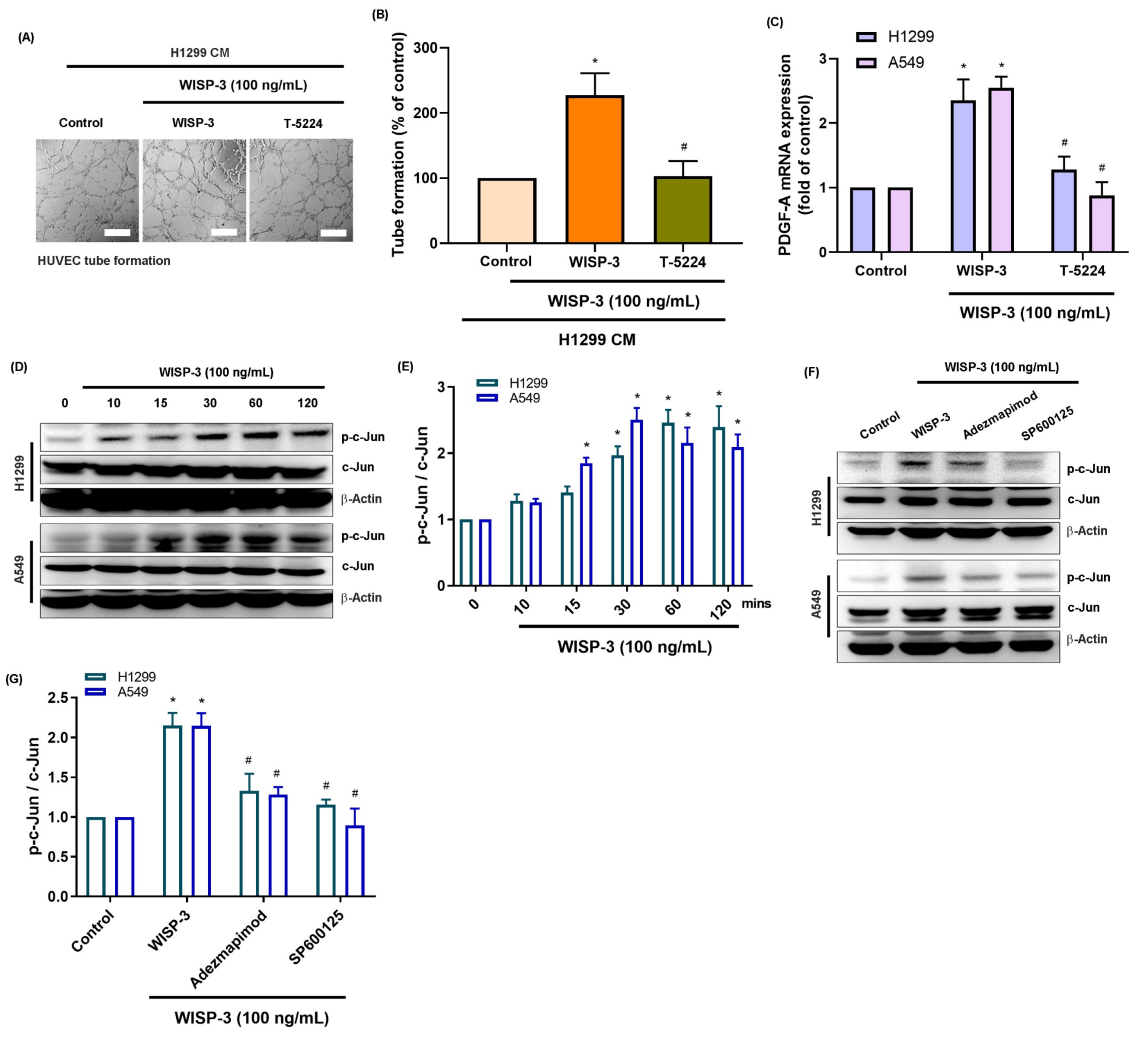
** WISP-3 activates c-Jun through p38 and JNK signaling to regulate PDGF-A expression and HUVEC tube formation.** (A) Representative micrographs of HUVEC tube formation assays showing the inhibitory effect of c-Jun inhibitor T-5224 on WISP-3-induced angiogenesis. (B) Quantitative analysis of total tube branch numbers relative to the control group. (C) PDGFA mRNA expression in H1299 and A549 cells treated with WISP-3 (100 ng/mL) in the presence or absence of the indicated inhibitors (Adezmapimod, SP600125, or T-5224). (D) Western blot analysis of time-dependent c-Jun phosphorylation (0-120 min) in LUAD cells following WISP-3 stimulation. (E) Densitometric quantification of p-c-Jun/c-Jun ratios normalized to the control group. (F&G) Western blot and densitometric analysis demonstrating that pharmacological inhibition of either p38 (Adezmapimod) or JNK (SP600125) significantly decreases WISP-3-induced c-Jun phosphorylation in both cell lines. Results are expressed as mean ± SD (n ≥ 3). *p < 0.05 vs. control; #p < 0.05 vs. WISP-3. Scale bar = 200 μm (for all micrographs).

**Figure 5:**
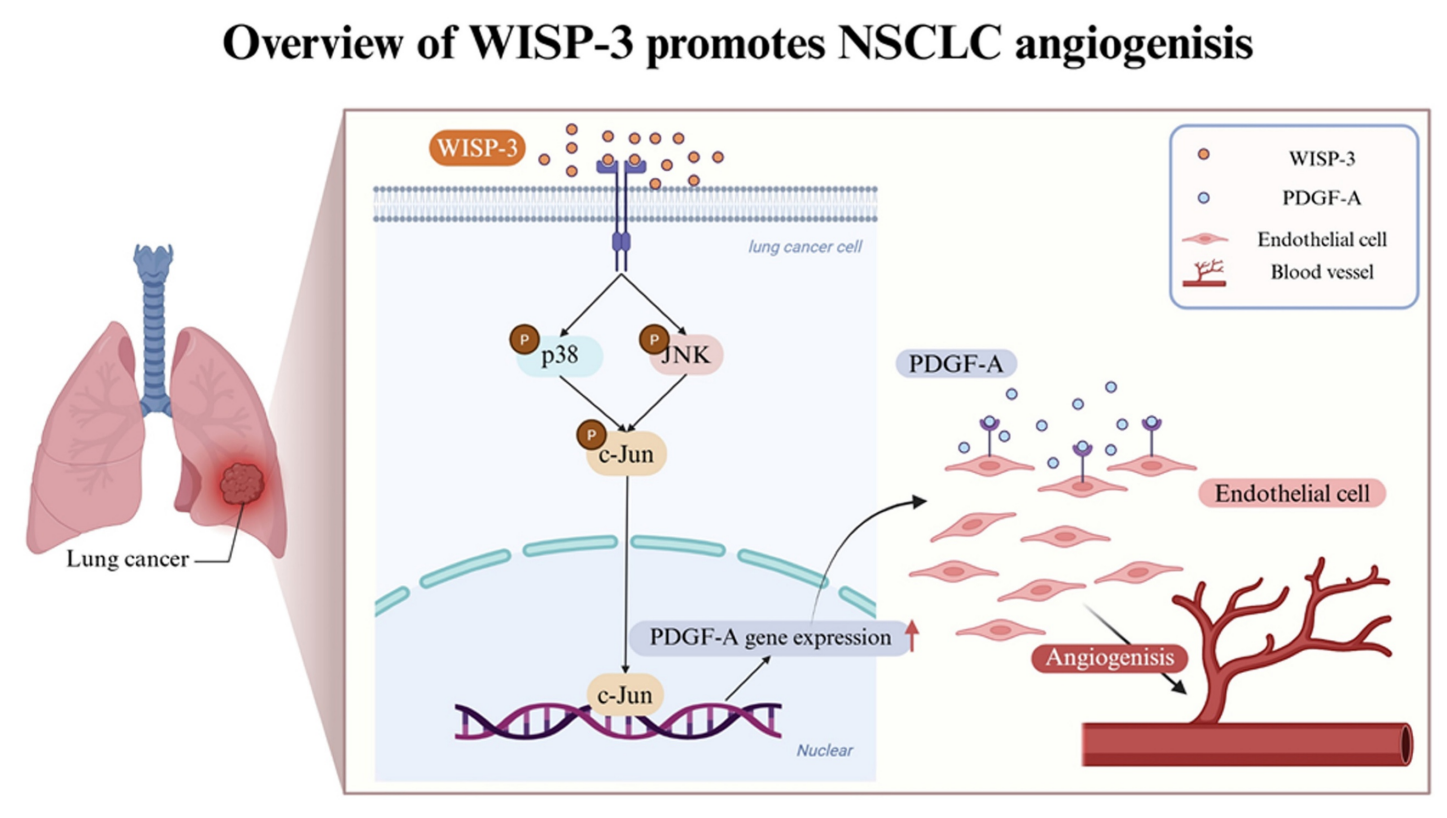
** Schematic illustration of WISP-3-mediated PDGF-A upregulation and angiogenesis in LUAD.** WISP-3 binds to its potential receptors on lung cancer cells, triggering the activation of the p38 and JNK signaling pathways. These cascades converge to induce the phosphorylation of the transcription factor c-Jun, which subsequently translocates to the nucleus (as part of the AP-1 complex) to promote PDGF-A gene expression. The secreted PDGF-A then acts in a paracrine manner on endothelial cells to drive tube formation, ultimately leading to enhanced angiogenesis within the lung adenocarcinoma microenvironment.

The transcription factor c-Jun, a downstream effector of the MAPK family and a core component of the AP-1 complex, regulates several angiogenic genes, including PDGF-A and VEGF-A 22. Given our observation that WISP-3 activates both p38 and JNK pathways, we investigated whether these kinases converge on c-Jun to mediate WISP-3-induced PDGF-A expression and angiogenesis. Our results showed that while recombinant WISP-3 (100 ng/mL) markedly enhanced HUVEC tube formation, pharmacological inhibition of c-Jun using T-5224 significantly abrogated this effect (Fig. 4A&B). Consistently, the WISP-3-induced upregulation of PDGF-A mRNA in both H1299 and A549 cells was effectively attenuated by c-Jun inhibitor (Fig. 4C). Western blot analysis revealed that WISP-3 rapidly triggered c-Jun phosphorylation in a time-dependent manner, peaking at 30-60 min and remaining sustained for up to 120 min (Fig. 4D&E). Notably, the inhibition of either p38 or JNK markedly suppressed WISP-3-induced c-Jun phosphorylation, confirming that c-Jun activation is a downstream event of both signaling cascades (Fig. 4F&G). These findings demonstrate that WISP-3 activates c-Jun through convergent p38 and JNK signaling, leading to the transcriptional upregulation of PDGF-A and the promotion of angiogenic activity in LUAD cells. This identifies c-Jun as a central signaling node linking WISP-3/MAPK activation to PDGF-A-mediated angiogenesis. Collectively, our data reveal a novel WISP-3/p38-JNK-c-Jun-PDGF-A signaling axis that promotes angiogenesis in non-small cell lung cancer, suggesting that targeting this pathway may provide a new therapeutic strategy for inhibiting tumor vascularization in LUAD (Fig. 5).

## Discussion

The CCN family of matricellular proteins has emerged as a critical regulator of tumor progression, ECM remodeling, and angiogenesis 23, 24. Among them, WISP-3(CCN6) is implicated in diverse biological processes, including cell proliferation, differentiation, and motility, largely through the modulation of MAPK and TGF-β signaling pathways 19, 23. However, its specific contribution to the angiogenic phenotype of LUAD has remained undefined. In the present study, we demonstrated that WISP-3 promotes angiogenesis in LUAD by activating the p38 and JNK MAPK signaling cascades, leading to c-Jun phosphorylation and subsequent transcriptional upregulation of PDGF-A. The secreted PDGF-A then enhances endothelial cell proliferation and tube formation, ultimately facilitating vascular remodeling within the LUAD tumor microenvironment. These findings identify a novel WISP-3/p38-JNK-c-Jun-PDGF-A signaling axis, linking WISP-3 activity to PDGF-A-driven angiogenesis in LUAD.

Angiogenesis is a hallmark of LUAD progression, and the PDGF family plays a pivotal role in tumor vascular development and pericyte recruitment 25, 26. High PDGF-A expression and activation of PDGFRα have been correlated with increased microvessel density and a poor clinical outcome in NSCLC 27, 28. Our findings extend these observations by establishing that WISP-3 functions as an upstream regulator of PDGF-A, providing mechanistic evidence for the involvement of the CCN family in PDGF-mediated angiogenesis. The inhibition of either p38 or JNK markedly attenuated WISP-3-induced PDGF-A expression and tube formation, underscoring the central role of the MAPK pathway in mediating WISP-3-dependent vascular remodeling in LUAD.

While WISP-3 has been extensively studied in other malignancies, such as breast cancer, where loss of WISP-3 expression is associated with aggressive tumor behavior and poor prognosis 29, its role in lung adenocarcinoma has remained underexplored. A recent bioinformatics analysis by Shi et al. (2024) identified WISP-3 as a hub gene upregulated in LUAD, correlated with alterations in the immune microenvironment, and associated with unfavorable survival outcomes 20. Our study provides the first functional validation of this prediction, demonstrating that WISP-3 actively contributes to vascular remodeling in LUAD by upregulating PDGF-A. By linking WISP-3 to endothelial activation and angiogenesis, our findings enhance the understanding of how stromal-tumor interactions mediated by CCN family proteins influence LUAD progression.

A key finding of this study is the identification of the p38 and JNK pathways as the primary drivers of WISP-3-induced PDGF-A expression. While the Mitogen-activated Protein Kinase (MAPK) family also includes the ERK1/2 pathway, which is frequently involved in growth factor signaling, our preliminary screening indicated that pharmacological inhibition of ERK1/2 did not significantly attenuate WISP-3-induced PDGF-A upregulation in our LUAD models (data not shown). This suggests a high degree of signaling specificity, where WISP-3 preferentially engages the stress-activated protein kinases (p38 and JNK) rather than the classical mitogenic ERK pathway to activate c-Jun and the AP-1 complex 28, 30. Despite these insights, several limitations of the present study should be acknowledged. First, while we utilized two distinct LUAD cell lines (A549 and H1299) to ensure reproducibility, the pro-angiogenic effects of WISP-3 were primarily evaluated using *in vitro* HUVEC tube formation assays. Future studies utilizing *in vivo* models, such as the chick chorioallantoic membrane (CAM) assay or xenograft mouse models, are necessary to validate the physiological relevance of WISP-3-mediated neovascularization. Second, although we focused on tumor-derived PDGF-A, the potential crosstalk between WISP-3 and other components of the TME, such as cancer-associated fibroblasts (CAFs) and immune cells, remains to be elucidated 31.

In summary, our study delineates a novel signaling axis wherein WISP-3 activates p38 and JNK, leading to c-Jun-dependent transcriptional upregulation of PDGF-A and subsequent promotion of angiogenesis. These findings highlight WISP-3 as a crucial regulator of the LUAD angiogenic landscape and a promising target for future anti-vascular therapeutic interventions.

## Figures and Tables

**Table 1:** List of RT-qPCR primers used in the experiments

Target mRNA	Forward primer (5'→3')	Reverse primer (5'→3')
PDGF-A	GCAAGACCAGGACGGTCATTT	GGCACTTGACACTGCTCGT
PDGF-B	CTCGATCCGCTCCTTTGATGA	CGTTGGTGCGGTCTATGAG
PDGF-C	ATTTGGGCTTGAAGACCCAGA	CCAGCGCCCTAATATAGTTCCA
PDGF-D	ACGGATACAGCTAGTGTTTGACA	GTCCACACCATCGTCCTCTAATA
VEGF-A	AGGGCAGAATCATCACGAAGT	AGGGTCTCGATTGGATGGCA
GAPDH	ACCACAGTCCATGCCATCAC	TCCACCACCCTGTTGCTGTA

## Data Availability

This published article includes all data generated or analyzed during this study. The data generated in this study are available upon reasonable request from the corresponding author.
